# Polymorphism and epitope sharing between the alleles of merozoite surface protein-1 of *Plasmodium falciparum *among Indian isolates

**DOI:** 10.1186/1475-2875-6-95

**Published:** 2007-07-20

**Authors:** Anitha Mamillapalli, Sujatha Sunil, Suraksha S Diwan, Surya K Sharma, Prajesh K Tyagi, Tridibes Adak, Hema Joshi, Pawan Malhotra

**Affiliations:** 1 Department of Parasitolgy, National Institute for Malaria Research, New Delhi, India; 2 Malaria Group, Internationl Centre for Genetic Engineering and Biotechnology, New Delhi, India; 3 presently working at Centre for Cellular and Molecular Biology, Hyderabad, India

## Abstract

**Background:**

The C-terminal region of merozoite surface protein-1 (MSP-1) is one of the leading candidates for vaccination against the erythrocytic stages of malaria. However, a major concern in the development of MSP-1 based malaria vaccine is the polymorphism observed in different geographical *Plasmodium falciparum *isolates. To explore whether the sequence heterogeneity of PfMSP-1 leads to variation in naturally acquired anti-MSP-1_19 _antibodies, the present study was undertaken to study PfMSP-1_19 _sequence polymorphism in malaria-endemic villages in eastern India and also carried out a competition enzyme-linked immunosorbent assay using three PfMSP-1_19 _variant forms.

**Methods:**

The sequence variations in the C-terminal region of PfMSP-1_19 _were determined in a malaria endemic region. Three PfMSP-1_19 _variants were produced in *Escherichia coli *(PfMSP1_19_QKNG-L, PfMSP1_19_EKNG-L and PfMSP1_19_ETSR-F) and an immunodepletion assay was carried out using the corresponding patients' sera.

**Results:**

Results revealed predominance of PfMAD20 allele among Indian field isolates. Seven PfMSP-1_19 _variant forms were isolated in a singe geographical location. Three of PfMSP-1_19 _variant forms when expressed in *E. coli *showed presence of cross-reaction as well as variant specific antibodies in malaria infected patient sera.

**Conclusion:**

The present study demonstrates the existence of allele specific antibodies in *P. falciparum*-infected patient sera, however their role in protection requires further investigation. These results thereby, suggest the importance of a multi-allelic PfMSP-1_19 _based vaccine for an effective malaria control.

## Background

Malaria is one of the major causes of death from infection in developing countries. Development of an effective malaria vaccine may reduce malaria-associated severe morbidity and mortality in malaria-endemic areas. A number of parasite surface antigens of asexual blood stages are being investigated as vaccine candidate antigens. [1,2]. Among these antigens, merozoite surface protein-1 (MSP-1) is a leading candidate antigen [3]. The *msp*-gene encodes a 195 kDa protein that is cleaved in four distinct fragments (83, 28–30, 38–45 and 42 kDa) at the time of schizont rupture. During merozoite invasion, the carboxy-terminal 42-kDa fragment is further processed to yield a 19-kDa fragment (MSP-1_19_) which remains associated with merozoites [4,5]. A number of vaccination studies with MSP-1_19 _and MSP-1_42_, in mice and monkeys have shown partial and full protection from malaria infection [6-11]. A substantial proportion of antibodies directed to MSP-1_19 _in *Plasmodium falciparum*-infected human sera have been shown to inhibit erythrocyte invasion *in vitro *[12]. Importantly, MSP-1_19_-mediated protective immune responses are largely antibody dependent with high antibody titres being essential for the protection [13,14].

The *msp-1 *of *P. falciparum *has been shown to be dimorphic, K1/Wellcome and MAD 20 types [15,16]. Sequence comparison of *P. falciparum msp-1 *sequences among different geographical isolates shows a great deal of variations. Based on sequence analysis, *msp-1 *has been divided into 17 blocks comprising of conserved, semi conserved and variable regions [5,15]. Intragenic recombination between the two allelic types appears to be the main cause for variability among different field isolates [16-18]. The C-terminal 19 kDa region of *msp-1*, that represents the 17^th ^block, consists of two EGF like domains [5] and has been shown to be highly conserved among different isolates, with single amino acid substitution at five different positions. These changes are E μ Q at position 1644 in the first EGF domain and at positions, 1691 (T μ K), 1700 (S μ N), 1701 (R μ G) and 1716 (L μ F) [19]. Based on these variations, several variant forms of PfMSP-1_19 _have been described among different *P. falciparum *isolates around the world. However, there are a limited number of reports of genetic diversity of C-terminal region of MSP-1_19 _in isolates from the Indian subcontinent [18,20,21], that contribute around two million cases every year (Source: National Vector Borne Disease Control Programme).

The present study investigated sequence variations in MSP-1_19 _region among different field isolates from a malaria endemic area in India. This study reveals existence of seven variant types in a single geographical location. In addition, three of the MSP-1_19 _variants, Q-KNG-L, E-KNG-L and E-TSR-F were expressed and the relative abundance of specific antibodies in their respective sera studied.

## Methods

### Collection of *P. falciparum *infected blood and sera

*P. falciparum *infected blood samples were collected on filter paper by finger prick from malaria patients participating in cross-sectional and longitudinal malaria epidemiology surveys being conducted in malaria endemic villages in Sundergarh district, Orissa in eastern India [23]. Thirteen study villages were chosen, out of which eight villages were located in deep forests and five villages were located in plain areas. Prior consent of the patients and the consent of institutional ethical committee were taken before the beginning of the study. Age of the patients ranged between six months to 17 years. In case of infants, the consent of parents were obtained. All the samples were positive for *P. falciparum *infection as determined by Giemsa-stained thick smears examined microscopically. Sixteen field spots were chosen from six different villages and DNA was isolated from these spots by the boiling method [24]. Blood samples were also collected and allowed to clot at room temperature, and serum was collected by centrifugation at 2,000 rpm for 10 min at 4°C and stored at -20°C until used.

### PCR amplification, cloning and sequencing

To determine the sequence polymorphism in PfMSP-1_19 _gene, PCR amplification was done in a 50 μl reaction volume using 10 ng genomic DNA from different *P. falciparum *isolates and primers MSP1-19F1 (Forward: 5' ATT GAG ACC TTA TAC AAT AAC 3') and MSP1-19R1 (Reverse: 5' TTA AGG TAA CAT ATT TTA ACT CCT AC 3'). The thermocycler profile was 5 min hot start at 94°C and 30 cycles each of 30 sec at 94°C, 1 min at 50°C and 30 sec at 72°C. In some cases, nested PCR was employed using a second set of forward primer MSP1-19F2 (Forward: 5' GAT ACG AAA AAA GAT ATG CTT GG 3') and the same reverse primer. The amplified PCR products (480 bps) were analysed by agarose gel electrophoresis and purified by QIAquick Gel Extraction Kit (Qiagen). The purified PCR fragments were cloned into pGEM-T cloning vector as per manufacturer's instructions (Promega). The positive clones were selected by restriction enzyme analysis. Three independent clones from a minimum of two PCR amplifications for each isolate were sequenced using T7 forward and M13 reverse primers.

### Expression and purification of three PfMSP-1_19 _variants

For the expression of PfMSP-1_19 _variants, the 288-bp fragment corresponding to PfMSP-1_19 _region were amplified using primers; 5'-GACTAGGGATCCATTTCACAACACCAATGCG-3' and 5'-GCTGATGTCGACTTAGTTAGAGGAACTGCAGAA-3' and cloned into pQE-30 expression vector in *Bam*HI and *Sal*I sites. This vector provides six His residues at the N terminus of the expressed protein. Expression of the recombinant MSP-1_19 _was induced with 0.5 mM IPTG in the *Escherichia coli *strain M15. For the purification of MSP-1_19 _proteins, the induced pellets were sonicated in 1 × TBS (Tris 50 mM, NaCl 500 mM), pH 8.0 containing 1 mM PMSF and 0.05% Tween 20. The supernatant containing the expressed proteins were incubated with Nickel-NTA-Agarose for 1 h at room temperature. The resins were washed with 1 × TBS containing 10 mM imidazole. The bound proteins were eluted with an imidazole gradient of 20 to 250 mM at pH 8.0. Q-KNG-L variant was eluted with a linear gradient of 10 to 70 mM Imidazole in 20 mM Tris-500 mM NaCl (pH 8.0) buffer, E-KNG-L variant was eluted in a linear gradient of 50–250 mM and E-TSR-F was eluted in 250 mM Imidazole. The eluates were analysed by SDS-PAGE, the fractions containing a clear single protein band were pooled, and the protein concentrations were determined.

### ELISA

Reactivity of the recombinant PfMSP-1_19 _proteins with patient sera was evaluated by ELISA as described earlier [25]. Briefly, 96-well microplates (Dynatech) were coated with 50 ng of any of the three recombinant PfMSP-1_19 _proteins per well in 0.06 M carbonate-bicarbonate buffer (pH 9.6). The plates were incubated overnight at 4°C, and the wells were blocked with 5% low-fat milk in PBS (pH 7.2) for 1 h at room temperature. The antigen-coated wells were sequentially incubated with serial dilutions (starting from 1:50 dilutions) of the individual patient sera and optimally diluted enzyme-labelled secondary antibody (horseradish peroxidase-labeled antihuman immunoglobulin, IgG). In between these incubations, the plates were washed with a 0.05% solution of Tween 20 in PBS. The enzyme reaction was developed with o-phenylediamine dihydrochloride-H_2_O_2 _in citrate phosphate buffer (pH 5.0), stopped with 8 N H_2_SO_4_, and recorded at 490 nm by use of a microplate reader (Molecular Devices).

### Antibody depletion assay

To determine the relative abundances of antibodies specific for a PfMSP1_19 _variant in *P. falciparum*-infected human sera, an antibody depletion assay was carried out as described earlier [25]. The wells of flat-bottom Immunolon-2 plates were coated with 100 ng of each of the PfMSP-1_19 _recombinant antigens, namely PfMSP1_19_QKNG-L, PfMSP1_19_EKNG-L and PfMSP1_19_ETSR-F. The wells were blocked with 5% low-fat milk in PBS (pH 7.2) for 1 h at room temperature. After blocking, all of the wells were washed with 0.05% Tween 20 in PBS and then with PBS (pH 7.2), the first two wells in the first column were incubated for ten minutes with *P. falciparum*-infected human sera of the respective sequence variants at a dilution of 1:100, while the remaining wells contained only wash buffer. The sera from the first two wells were transferred to the next respective wells in the second column and incubated for half an hour. These serial incubations were carried out until all of the antibodies with respect to a particular antigen were depleted, as determined by color development in the wells by a standard ELISA. It was observed that for all the sequence variantss, antigen-specific antibodies were completely removed from the respective sera after six serial transfers except in the case of Q-KNG-L variant which depleted the sera after nine serial transfers. All these antibody-depleted sera were subsequently analysed for their cross reactivity with each of the PfMSP1_19 _variants; PfMSP1_19_Q-KNG-L, E-KNG-L and PfMSP1_19_E-TSR-F antigens by ELISA. The reactivity of each protein with the other two depleted sera was compared with the undepleted sera to analyse the relative contribution of each antigen.

## Results

### Epidemiology of malaria transmission in malaria endemic villages in Orrisa, India

Thirteen study villages were chosen, out of which eight villages were located in deep forests and five villages were located in plain areas. A detailed study of the epidemiology of malaria transmission was done in these villages from January 2001 to December 2003 and was reported [26]. Briefly, malaria transmission is perennial but is markedly low in the plain area than forest area. *Plasmodium falciparum *accounted for 85% of total malaria cases during the study period. In forest and plain areas, the number of *P. falciparum *cases per thousand populations were 284.1 and 31.2 respectively, whereas the parasite rate was 14% and 1.7% respectively. In forest areas, clinical malaria occurred more frequently in children aged 0–5 years and declined gradually with increasing age. *Anopheles fluvialitis *and *Anopheles culicifacies *were the two vector species responsible for malaria transmission in these villages. The entomological inoculation rate in the forest and plain areas was 0.311 and 0.014 infective bites/person/night, respectively during 2003. The peak of malaria incidence coincided with peak vector density of *A. fluvialitis*, suggesting that this is the predominant vector for malaria transmission in these villages. Parasite density with a geo mean density of more than 2000 were detected in 211 patients in the forest area as compared to 28 patients in the plain area.

### Sequence polymorphism in PfMSP-1_19 _gene

**Figure 1:**
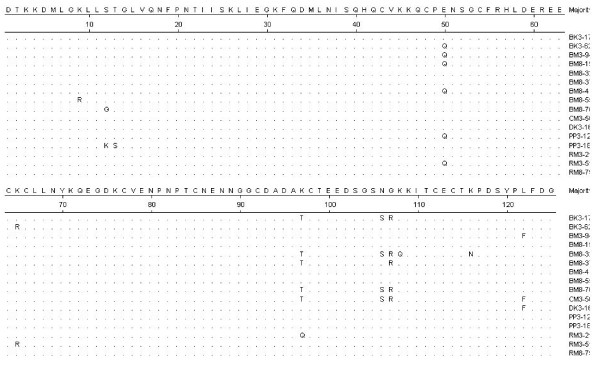
Sequence diversity in the C-terminal region (blocks 16 and 17) of MSP-1 in Indian *P. falciparum *isolates in a single geographical location.

To determine the extent of polymorphism of MSP-1_19 _in a natural population of *P. falciparum *in India, the C-terminal region of MSP-1_19 _gene corresponding to 16^th ^block (a variable region) and 17th block (a conserved region) was amplified from 16 *P. falciparum *isolates. The amplified fragments were sequenced and these sequences were compared with that of two prototypic alleles, MAD20 and K1/Wellcome (Figure 1). Comparison of the sequences between 1594 and 1630 aa that correspond to the 3' end of 16^th ^block, showed that all these field isolates belong to one of the two prototypic alleles, MAD20. Existence of both allelic forms in a single isolate was not observed. All the sequences corresponding to 16^th ^block were highly conserved; only a few variations were seen in two of the 16 isolates. A change K μ R was observed at amino acid position 1603 in one of the isolate, BM8-55. These results suggested the predominance of MAD 20 allelic type among these field isolates in India.

In block 17 which represents the C-terminal, cysteine-rich 19 kDa fragment of MSP-1 (1631–1744), the nucleotide and the deduced amino acid sequences were found to be highly conserved with only five substitutions seen at positions, 1644 (E μ Q), 1691 (T μ K), 1700 (S μ N), 1701 (G μ R) and 1716 (L μ F) among Indian isolates (Fig. 1). All the nucleotide changes in these isolates were non-synonymous, as a result, the deduced amino acid variation corresponded to one or other allele. Based on these five changes, seven different allelic forms were identified, many of which have been reported earlier [19]. Among the several alleles of PfMSP-1_19_, Q-KNG-L allele was seen in 5/16 isolates, while E-KNG-L and E-TSR-L alleles were observed in 3/16 isolates. Existence of two allelic forms in a single patient was not observed. Table 1 shows the distribution of different allelic types among theses Indian isolates. In addition to the reported combinations of the allele determining residues, we observed two novel allelic forms, E-TSR-F and E-TNR-L that have been predicted earlier [19]. Few other substitutions was seen in one of the isolate BM8-32, at positions 1701 (K μ Q) and 1710 (K μ N). Changes as reported in other geographical locations [19,27] were not observed among these Indian isolates.

**Table 1:** Distribution of PfMSP-1_19 _allelic types identified in Indian *P. falciparum *isolates.

S.No	ISOLATE	TYPE	SEQUENCE
1.	BK3-17	MAD20	E-TSR-L
2.	BK3-62	WELLCOME	Q-KNG-L
3.	BM3-94	THAI	Q-KNG-F
4.	BM8-19	WELLCOME	Q-KNG-L
5.	BM8-32	MAD20	E-TSR-L
6.	BM8-37	NOVEL	E-TNR-L
7.	BM8-4	WELLCOME	Q-KNG-L
8.	BM8-55	UGANDA	E-KNG-L
9.	BM8-70	MAD20	E-TSR-L
10.	CM3-56	NOVEL	E-TSR-F
11.	DK3-16	UGANDA	E-KNG-F
12.	PP3-12	WELLCOME	Q-KNG-L
13.	PP3-18	UGANDA	E-KNG-L
14.	RM3-21	UGANDA	E-KNG-L
15.	RM3-51	WELLCOME	Q-KNG-L
16.	RM8-78	UGANDA	E-KNG-L

### Expression of three PfMSP-1_19 _variants and epitope sharing among these isolates

**Figure 2:**
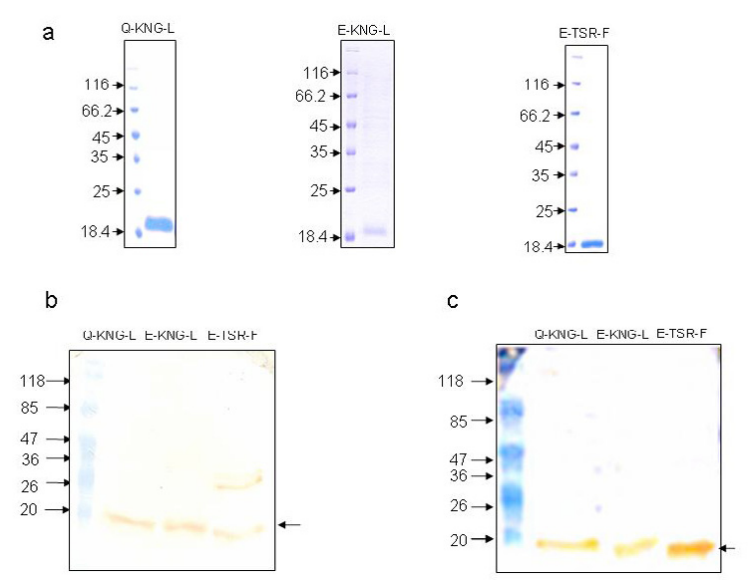
Expression and immunoblot analysis of *E. coli *expressed PfMSP-1_19 _variants. (a) Coomassie blue-stained 12% SDS-polyacrylamide gel of purified PfMSP-1_19 _variants. (b & c) Immunoblot analysis to show the reactivity of PfMSP-1_19 _variants with monoclonal antibodies (12.10) and with anti-PfMSP1_19 _(Q-KNG-L) antibody.

The sequenced PfMSP-1_19 _gene fragment corresponding to three variant types (Q-KNG-L, E-KNG-L and E-TSR-F) isolated from *P. falciparum *infected patients (BK-62, PP3-18 and CM3-56) were cloned into pQE-30 vector and expression was induced with IPTG. All the three PfMSP-1_19 _variants were expressed in soluble form and the proteins were purified up to 85% by Ni-NTA^2+ ^chromatography. The three variant forms of PfMSP-1_19 _migrated as a single major band on SDS-PAGE under reducing conditions and had apparent molecular mass of ~17–19 kDa (Figure 2A). *Escherichia coli*-expressed PfMSP-1_19 _variants were recognized by monoclonal antibody, 12.10 and a polyclonal antibody raised against Q-KNG-L variant type (Figures 2B and 2C). These results were in accordance with previous findings [28].

**Figure 3:**
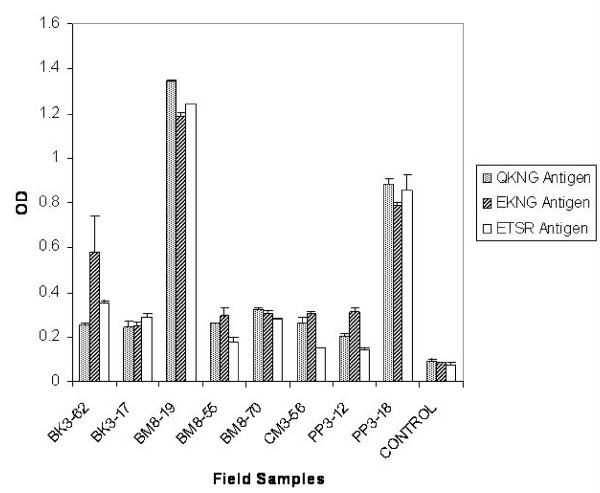
Reactivity of PfMSP-1_19 _variants in an ELISA with sera collected from *P. falciparum *infected patients at a dilution of 1:200. Error bars indicate S.D.

**Figure 4:**
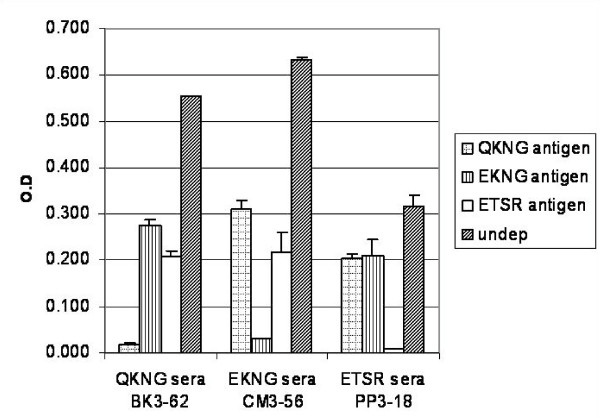
Immunodepletion assay showing the presence of PfMSP1_19 _allele specific antibodies in sera from *P. falciparum *infected patients. Error bars indicate S.D.

To examine the cross-reactive epitopes in the three variant types, an ELISA assay was carried out using their respective sera and with sera obtained from patients showing other variant types. As shown in Figure 3, the three variant types showed reactivity with each sera; however the extent of reactivity varied with the patient sera. To determine the contribution of variant specific epitopes in the generation of an immune response during a natural infection, an immunodepletion assay was carried out. For the ELISA depletion assay, sera from *P. falciparum*-infected patients were absorbed with either of the variant recombinant proteins in such a way that all of the antigen specific antibodies were lost. The reactivity of the sera depleted was further analysed with all the expressed PfMSP-1_19 _variant forms. As shown in Figure 4, when the sera from patient, BK3-62 expressing Q-KNG-L variant type was depleted with either Q-KNG-L or E-KNG-L or E-TSR-F proteins, there was < 90% reduction in reactivity of the depleted sera with Q-KNG-L antigen in comparison to its reactivity with undepleted sera. These depleted sera also showed a reduction of ~40–50% in its reactivity to other two PfMSP-1_19 _variant types. Likewise, when sera from patients, PP3-18 expressing E-KNG-L variant type and CM3-56 expressing E-TSR-F was depleted with either of the three PfMSP-1_19 _variant forms, similar reduction was seen. These results showed that PfMSP-1_19 _variant specific immune responses are generated in *P. falciparum *infected patients.

## Discussion

Sequence heterogeneity in PfMSP-1_19 _protein, a leading vaccine candidate antigen for vaccination against erythrocytic stages of *P. falciparum*, may compromise its use as a vaccine candidate antigen. Based on sequence variations among different laboratory and field isolates, two prototypic alleles of *P. falciparum *MSP-1 represented by PfMAD20 and PfK1/Wellcome have been described in isolates from Africa, Asia and Latin America [15,16,24,25]. The C-terminal 19 kDa region of MSP-1 (PfMSP-1_19_) is a highly conserved region showing amino-acid alterations at only five positions out of 102 residues. Based on these alterations, about ten different PfMSP-1_19 _allelic forms have been predicted in different field isolates across the world [19].

Here, sequence diversity in MSP-1_19 _region, among Indian *P. falciparum *field isolates in malaria endemic villages in same geographical location was investigated. Some of these isolates have been previously analysed for sequence variations in the receptor binding domain of *Pf*EBA-175 [29]. Sequence data of the 16^th ^block of MSP-1 revealed that all the isolates were of PfMAD20 type. The present study's results and the data obtained from previous studies clearly demonstrate that MAD20 allelic form predominates among Indian PfMSP-1 isolates from different geographical locations [18,20,30]. In the 17^th ^block, seven allelic forms was observed. Of these PfMSP-1_19 _alleles, two alleles (E-TNR-L & E-TSR-F) have not been reported earlier, however, Qari et al (1998) have earlier predicted E-TNR-L. These allelic forms seem to have generated due to intragenic recombination between the two prototypic alleles as suggested earlier because all the substituted amino acids belonged to either one or other allelic form.

To know whether these five amino acid substitutions in the PfMSP-1_19 _region generate variant specific immune responses in *P. falciparum *infected patients, three PfMSP-1_19 _variants (Q-KNG-L, E-KNG-L & E-TSR-F) were expressed in an *E. coli *expression system with an N-terminal His tag. Proteins were purified on a Ni^2+^-NTA column. All the three PfMSP-1_19 _recombinant forms were recognized well by an invasion inhibitory monoclonal antibody (12.10) and anti-PfMSP-1_19 _(Q-KNG-L) polyclonal antibody. An earlier study [28] showed that the yeast secreted recombinant MSP-1_19 _variants are recognized by polyclonal antibody raised against baculoproduced MSP1 (E-KNG) variant and by mAB 5B1 that is known to inhibit invasion. Recognition of all the PfMSP-1_19 _variants by mAB 5B1 and 12.10 suggest that some of these protective epitopes are not variant specific. A preliminary investigation of protective type IgG1/IgG3 antibody response to Q-KNG-L sequence variant was carried out and significant IgG1/IgG3 responses were observed in the infected sera (data not shown). The frequency of IgG1 responses in different sera was quite similar while a significant difference was seen within IgG3 responses as previously shown [31].

To determine the presence of variant specific antibodies during the natural infection, sera from BK3-62, CM3-56 and PP3-18 patients were immuno-depleted using each of the three recombinant antigens and the depleted sera were analysed for reactivity with three variant antigens. Results demonstrated that sera from *P. falciparum *infected patients do contain cross-reactive as well as PfMSP-1_19 _variant specific antibodies. At present, it is difficult to say up to what extent these variant specific antibodies contribute to protective immune responses. Another aspect that requires probing is the contribution of historic infections with parasite of different sequence types. However, data from a number of challenge studies have also provided evidences for the variant specific immune responses. Immunization of mice with recombinant PyMSP-1_19 _protected mice against homologous but not heterologous sporozoite or blood stage challenges [32,33]. In some cases where some level of protection to heterologous malarial challenge was seen, it was significantly lower than the homologous challenge [6,34].

## Conclusion

In summary, the present study has shown that PfMAD 20 is the major PfMSP-1 allelic form present in Indian population and a number of PfMSP-1_19 _allelic forms exist even in a single geographical location in a malaria-endemic region in India. The present study further show that significant levels of cross reactive antibodies are generated against different PfMSP-1_19 _allelic forms in a *P. falciparum *infected natural human population. However, presence of PfMSP-1_19 _allele specific antibodies was also observed in this population thus suggesting, for a better protection against all strains of *P. falciparum*, it will be appropriate to use a vaccine containing all possible sequence variants. Although only three PfMSP-1_19 _variants were expressed and subsequently analysed for the presence of specific antibodies in the present study, a detailed study is required to clearly understand the role of PfMSP-1_19 _variants in eliciting a protective immune response.

## Authors' contributions

AM carried out polymorphism studies, SS expressed the proteins and carried out the ELISAs and depletion assays. Both AM and SS contributed equally in the study. SSD participated in depletion assays, SKS was involved in sample collection, PKT participated in sample collection, TA provided with sera samples, HJ handled sequence data, PM participated in design and coordination of the project along with drafting the manuscript. All authors read and approved the final manuscript.

## References

[B1] Richie TL, Saul A (2002). Progress and challenges for malaria vaccines. Nature.

[B2] Sachs J, Malaney P (2002). The economic and social burden of malaria. Nature.

[B3] Good MF (2001). Towards a blood-stage vaccine for malaria: are we following all the leads?. Nat Rev Immunol.

[B4] Blackman MJ, Holder AA (1992). Secondary processing of the *Plasmodium falciparum *merozoite surface protein-1 (MSP1) by a calcium-dependent membrane-bound serine protease shedding of MSP133 as a noncovalently associated complex with other fragments of the MSP1. Mol Biochem Parasitol.

[B5] Cooper JA (1993). Merozoite surface antigen-1 of *Plasmodium*. Parasitol Today.

[B6] Singh S, Kennedy C, Long CA, Saul AJ, Miller LH, Stowers AW (2003). Biochemical and immunological characterization of bacterially expressed and refolded *Plasmodium falciparum *42-kilodalton C-terminal merozoite surface protein 1. Infect Immun.

[B7] Singh S, Miura K, Zhou H, Muratova O, Keegan B, Miles A, Martin LB, Saul AJ, Miller LH, Long CA (2006). Immunity to recombinant *Plasmodium falciparum *merozoite surface protein (MSP1): Protection in *Aotus nancymai *monkeys strongly correlates with anti-MSP1 antibody titer and in vitro parasite-inhibitory activity. Infect Immun.

[B8] Sachdeva S, Mohmmed A, Dasaradhi PVN, Crabb BS, Katyal A, Malhotra P, Chauhan VS (2006). Immunogenicity and protective efficacy of *Escherichia coli *expressed *Plasmodium falciparum *merozoite surface protein-142 using human compatible adjuvants. Vaccine.

[B9] Chang SP, Case SE, Gosnell A, Hashimoto KJ, Kramer LQ, Tam CQ, Hashiro CM, Nikaido HL, Gibson CT, Lee-Ng, Barr PJ, Yokota BT, Hui GSN (1996). A recombinant baculovirus 42-kilodalton C-terminal fragment of *Plasmodium falciparum *merozoite surface protein 2 protects *Aotus *monkeys against malaria. Infect Immun.

[B10] Tian JH, Kumar S, Kaslow DC, Miller LH (1997). Comparison of protection induced by immunization with recombinant proteins from different regions of merozoite surface protein 1 of *Plasmodium yoelii*. Infect Immun.

[B11] Egan A, Blackman MJ, Kaslow DC (2000). Vaccine efficacy of recombinant *Plasmodium falciparum *merozoite surface protein 1 in malaria-naïve, -exposed, and/or, -rechallenged *Aotus vociferans *monkeys. Infect Immun.

[B12] Egan A, Burghaus P, Druilhe A, Holder A, Riley E (1999). Human antibodies to the 19 kDa C-terminal fragment of *Plasmodium falciparum *merozoite protein 1 inhibit parasite growth *in vitro*. Parasite Immunol.

[B13] Daly TM, Long CM (1995). Humoral response to a carboxyl-terminal region of the merozoite surface protein 1 plays a predominant role in controlling blood-stage infection in rodent malaria. Infect Immun.

[B14] Hirunpetcharat C, Tian J, Kaslow D, van Rooijen N, Kumar S, Berzofsky J, Miller L, Good M (1997). Complete protective immunity induced in mice by immunization with the 19-kilodalton carboxyl-termianl fragment of the merozoite surface protein-1 (MSP[19]) of *Plasmodium yoelii *expressed in *Saccharomyces cerevisiae*: correlation of protection with antigen-specific antibody titer, but not with effector CD4+ T cells. J Immunol.

[B15] Tanabe KT, Mackay M, Goman M, Scaif JG (1987). Allelic dimorphism in a surface antigen gene of the malaria parasite *Plasmodium falciparum*. J Mol Biol.

[B16] Kang Y, Long CA (1995). Sequence heterogeneity of the C-terminal, Cys-rich region of the merozoite surface protein-1 (MSP-1) in field samples of *Plasmodium falciparum*. Mol Biochem Parasitol.

[B17] Cheng Q, Stowers A, Huang TY, Bustos D, Huang YM, Rzepckyk C, Saul A (1993). Polymorphism in *Plasmodium vivax *MSA1 gene- the result of intragenic recombinations?. Parasitol.

[B18] Lalitha PV, Malhotra P, Chattopadhyay R, Chauhan VS (1999). *Plasmodium falciparum *variations in the C-terminal cysteine rich region of the merozoite surface protein-1 field samples among Indian isolates. Exp Parasitol.

[B19] Qari SH, Ya-Ping Shi, Goldman IF, Nahlen BI, Tibaurenc M, Lal AA (1998). Predicted and observed alleles of *Plasmodium falciparum *merozoite surface protein (MSP-1) and a potential malaria vaccine antigen. Mol Biochem Parasitol.

[B20] Kumar S, Yadava A, Keister DB, Tain JH, Ohl KA (2005). Immunogenicity and in vivo efficacy of recombinant *Plasmodium falciparum *merozoite surface protein-1 in *Aotus *monkeys. Mol Med.

[B21] Raj DK, Das BR, Dash AP, Supakar C (2005). Genetic diversity in the merozoite surface protein 1 gene of *Plasmodium falciparum *in different malariaendemic localities. Am J Trop Med Hyg.

[B22] Sharma SK, Chattopadhyay R, Chakraborti K, Pati SS, Srivastava VK, Tyagi PK, Mahanty S, Misra SK, Adak T, Das BS, Chitnis CE (2004). Epidemiology of malaria transmission and development of natural immunity in a malaria endemic village, San Dulakudar, in Orissa state, India. Am J Trop Med Hyg.

[B23] Wooden J, Kyes S, Sibley CH (1993). PCR and strain identification in *Plasmodium falciparum*. Parasitol Today.

[B24] Sachdeva S, Gul Ahmad G, Malhotra P, Mukherjee P, Chauhan VS (2004). Comparison of immunogenicity of recombinant *Plasmodium vivax *merozoite surface protein 1 19- and 42- Kilodalton Fragments Expressed in *Escherichia coli*. Infect Immun.

[B25] Jongwutiwes S, Tanabe K, Kanbara H (1993). Sequence conservation in the Cterminal part of the precursor to the major merozoite surface proteins (MSP1) of *Plasmodium falciparum *from field isolates. Mol Biochem Parasitol.

[B26] Sharma SK, Tyagi PK, Padhan K, Upadhayay AK, HAque MA, Nanda N, Joshi H, Biswas S, Adak T, Das BS, Cahuhan VS, Chitnis CE, Subbarao SK (2006). Epimediology of malaria transmission in forest and plain ecotype villages in Sundargarh district, Orissa, India. Trans R Soc Trop Med Hyg.

[B27] Miller L, Toberts T, Shahabuddin M, McCutchan TF (1993). Analysis of sequence diversity in the *Plasmodium falciparum *merozoite surface protein-1 (MSP-1). Mol Biochem Parasitol.

[B28] Kaslow DC, Hui D, Kumar S (1994). Expression and antigenticity of *Plasmodium falciparum *major surface protein (MSP1_19_) variants secreted from *Saccharomyces cerevisiae*. Mol Biochem Parasitol.

[B29] Mamillapalli A, Pattnaik P, Sharma M, Sharma SK, Tyagi PJ, Joshi H, Chitnis CE (2006). Sequence polymorphisms in the receptor-binding domain of *Plasmodium falciparum *EBA-175: Implications for malaria vaccine development. Mol Biochem Parasitol.

[B30] Vijay Kumar S, Ranjan S, Saxena V, Rajesh V, Roy SK, Kochar D, Ranjan A, Das A (2005). *Plasmodium falciparum*: Genetic diversity of C-terminal region of MSP-1 in isolates from Indian sub-continent. Exp Parasitol.

[B31] Diallo TO, Spiegel A, Doiuf A, Perraut R, Kaslow DC, Garraud O (2001). IgG1/IgG3 antibody responses to various analogs of recombinant yPf MSP1_19_- a study in immune adults living in areas of *Plasmodium falciparum *transmission. Am J Trop Med Hyg.

[B32] Renia L, Ling IT, Marussig M, Miltgen F, Holder AA, Mazier D (1997). Immunization with recombinant C-terminal fragment of *Plasmodium yoelii *merozoite protein 1 protects mice against homologous but not heterologous *P. yoelii *sporozoite challenge. Infect Immun.

[B33] Rotamn HL, Daly TM, Long CC (1999). *Plasmodium*: immunization with carboxyl-terminal regions of MSP-1 protects against homologous but not heterologous blood-stage parasite challenge. Exp Parasitol.

[B34] Cox FE (1970). Protective immunity between malaria parasite and piroplasms in mice. Bull World Health Organ.

